# Functional interplay between long non-coding RNAs and the Wnt signaling cascade in osteosarcoma

**DOI:** 10.1186/s12935-021-02013-8

**Published:** 2021-06-15

**Authors:** Jieyu He, Lin Ling, Zhongyue Liu, Xiaolei Ren, Lu Wan, Chao Tu, Zhihong Li

**Affiliations:** 1 Department of Orthopedics, The Second Xiangya Hospital, Central South University, No 139 Middle Renmin Road, Changsha, 410011 Hunan China; 2 Hunan Key Laboratory of Tumor Models and Individualized Medicine, The Second Xiangya Hospital, Central South University, Changsha, 410011 Hunan China; 3 Department of Geriatrics, The Second Xiangya Hospital, Central South University, Changsha, 410011 Hunan China

**Keywords:** LncRNA, Wnt, β-catenin, Osteosarcoma, Drug resistance, Stemness

## Abstract

Osteosarcoma is a common and highly malignant bone tumor among children, adolescents and young adults. However, the underlying molecular mechanisms remain largely unexplored. LncRNAs are transcripts with no or limited protein-coding capacity in human genomes, and have been demonstrated to play crucial functions in initiation, progression, therapeutic resistance, recurrence and metastasis of tumor. Considerable studies revealed a dysregulated lncRNA expression pattern in osteosarcoma, which may act as oncogenes or suppressors to regulate osteosarcoma progression. Wnt signaling pathway is an important cascade in tumorigenesis by modulation of pleiotropic biological functions including cell proliferation, apoptosis, differentiation, stemness, genetic stability and chemoresistance. Hyperactivation or deficiency of key effectors in Wnt cascade is a common event in many osteosarcoma patients. Recently, increasing evidences have suggested that lncRNAs could interplay with component of Wnt pathway, and thereby contribute to osteosarcoma onset, progression and dissemination. In this review, we briefly summarize Wnt signaling-related lncRNAs in osteosarcoma progression, aiming to gain insights into their underlying crosstalk as well as clinical application in osteosarcoma therapeutic modalities.

## Introduction

Osteosarcoma is one of the most prevalent bone malignancies and account for an inordinate amount of tumor-related deaths in pediatric and adolescents patients [1, 2]. Currently, the standardized approaches are a combination of surgical resection and neoadjuvant/adjuvant chemotherapy [3]. Due to its high aggressiveness and risk of metastatic progression and recurrence after therapy, the prognosis of osteosarcoma is still dismal. Despite several advancements have been achieved in the multimodal treatment, the survival rate for osteosarcoma patients has reached a plateau. Currently, it is estimated that the 5-year survival rate is around 75% for localized osteosarcoma and only 30% in metastatic patients [4, 5]. Besides, the regulatory network in osteosarcoma is still obscure, and thus no targeted therapy could be implemented, posing a significant challenge to current modalities [6]. Therefore, it is a substantial need to explore the underlying molecular biology and pathogenesis in osteosarcoma progression and to develop corresponding novel therapies to improve the prognosis of patients [7, 8].

**Fig. 1:**
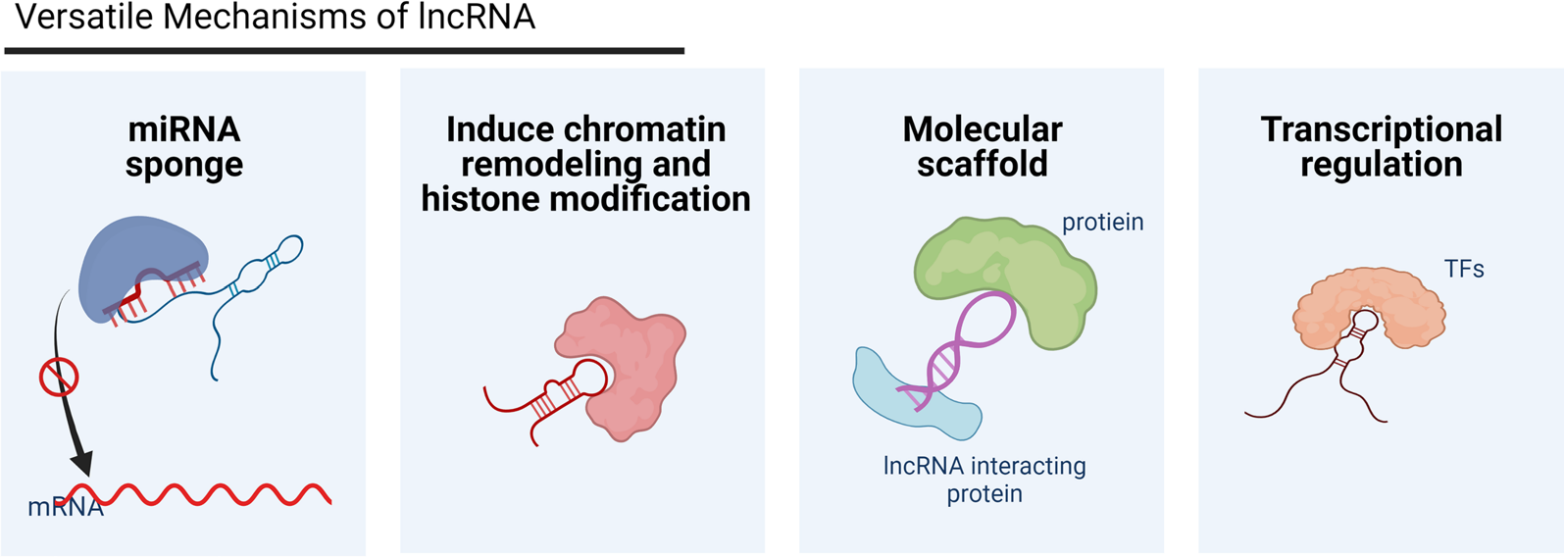
The schematic drawing shows partial regulatory mechanisms of action of lncRNA. Figure created with BioRender.com

Long non-coding RNAs (LncRNAs) refer to a cluster of non-coding RNAs (ncRNAs) that longer than 200 nucleotides, with no or limited protein-coding ability [9]. Due to rapid development of next-generation sequencing, accumulating lncRNA transcripts have been unveiled and annotated in recent years [10, 11]. Aberrant expression and dysregulation of lncRNAs has been implicated in the pathophysiology of a broad spectrum of human diseases, ranging from aging [12–14], neurodegenerative diseases [15], osteoarthritis [16], and cancers [17–20]. LncRNAs could participate in diverse biological processes, including cell survival, apoptosis, differentiation, DNA damage repair, and inflammation [21]. Mechanistically, the most commonly reported feature of lncRNAs is to act as a competing endogenous RNA (ceRNA) to regulate miRNA expression and thus to target downstream genes [22]. Besides, lncRNAs could also interact with macromolecules such as DNA, RNA and proteins to modulate expression of target genes at transcriptional, posttranscriptional and translational level, providing for multiple layers of control, as shown in Fig. 1 [21, 23]. Generally, the function of lncRNAs is location-specific [24]. For instance, the nucleus lncRNAs are usually engaged in chromatin remodeling, mRNA splicing, epigenetic regulation and phase separation [25], while those lncRNA in cytoplasm are involved in translational and post-translational modifications [23, 26].

Moreover, lncRNAs are reported to interplay with several important pathways in tumorigenesis, including Hippo [27], Notch [28], Hedgehog [29], PI3K/Akt [30], JAK/STAT [31] and Wnt [32], to exert a variety of effects in cellular processes. Of them, Wnt cascade is highly complex, while its function and cross-interplay with others remain largely unknown. Given its central and pleiotropic effects in cancer, including proliferation, cell fate specification and differentiation, and mitogenic stimulation, Wnt signaling has attracted much attention in recent decades. In osteosarcoma, lncRNAs play a regulatory role in the occurrence and development of osteosarcoma. Various lncRNAs exert promoting or inhibitory effects by targeting OS cell proliferation, invasion and migration via these above-mentioned oncogenic pathways. In addition, lncRNAs act in the ceRNA system to impact the lncRNAs-miRNAs interaction. More detailed mechanisms are involved in two recently-published reviews by Zhang et al. and Han et al. [1, 33]. There are several papers concerning roles of lncRNAs or Wnt/β-catenin pathway in osteosarcoma or crosstalk between lncRNAs and Wnt pathway with a few depictions of the intersection among these three [1, 6, 34]. However, the complex crosstalk and regulatory loop between lncRNAs and Wnt signaling pathway in osteosarcoma is still elusive, and have not been reviewed in details. In the context of this review, we summarized the most recent knowledge regarding the functional role of lncRNAs associated with Wnt signaling pathway, aiming to provide novel insights into understanding osteosarcoma pathogenesis and reveal a potential clinical application of Wnt signaling pathway-related lncRNAs in osteosarcoma.

## Wnt signaling pathway in tumorigenesis of osteosarcoma

Wnt signaling was firstly discovered in 1982, and is a highly evolutionarily conserved regulatory system [35, 36]. It has been identified to control multiple biological functions, including but limited to cell fate determination, differentiation, cell polarity, stem cell renewal, and mitogenic stimulation during embryonic development and tissue homeostasis [37, 38].

### Canonical and non-canonical Wnt signaling pathways

**Fig. 2:**
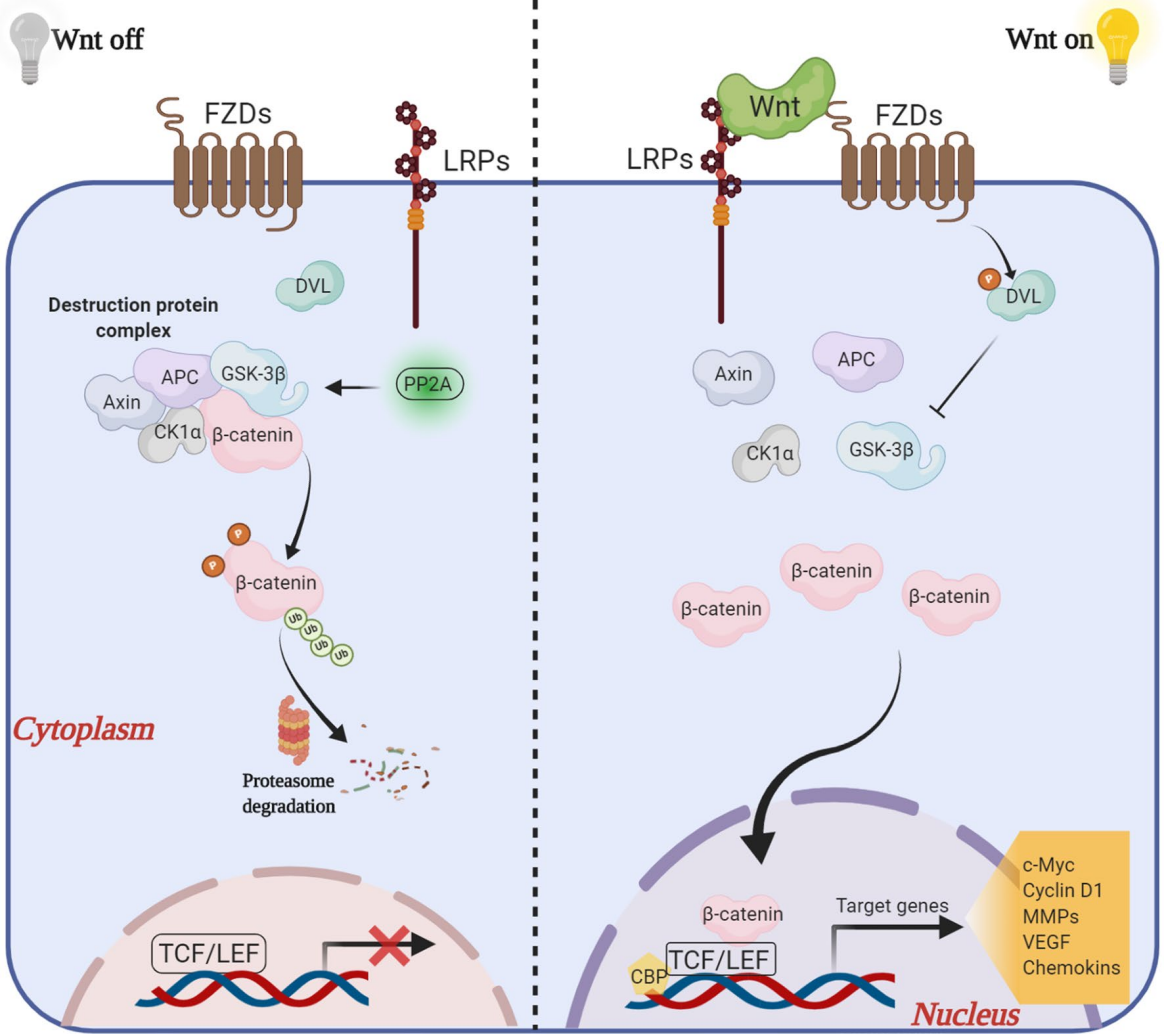
The schematic drawing demonstrates regulatory network of canonical Wnt signaling. Figure created with BioRender.com

The Wnt family consists of canonical and non-canonical pathways, which is β-catenin-dependent and β-catenin-independent, respectively [39]. Typically, the canonical pathway is best-characterized and its functions are driven by transcriptional co-activator β-catenin [40], as shown in details in Fig. 2. The Wnt proteins (secreted glycoproteins) couple with several receptors, such as Frizzleds (FZDs), low-density lipoprotein receptor-related proteins (LRPs) at the plasma membrane, to activate downstream pathways [40]. In the absence of extracellular Wnt (“Wnt off”), β-catenin is sequestrated, phosphorylated and subsequently degraded mediated by proteasome and tightly controlled by a “destruction protein complex” comprising adenomatous polyposis coli (APC), casein kinase 1α (CK1α), glycogen synthase kinase 3β (GSK-3β), protein phosphatase 2A (PP2A) and Axin that binds to β-catenin for destruction [41]. In contrast, upon stimulation by Wnt (“Wnt on”), the cytoplasmic dephosphorylated β-catenin is maintained, accumulated and translocated into nucleus, which thereby interacts with transcriptional factors T cell factor (TCF)/ lymphoid enhance factor (LEF) families, and CREB-binding protein (CBP) to initiate the expression of a large set of important developmental genes, such as c-Myc, Cyclin D1, matrix metalloproteinases (MMPs), vascular endothelial growth factors (VEGFs) and chemokines [42, 43].

Besides, the non-canonical branch functions in a β-catenin-independent manner. It can be further classified into two pathways, namely the Wnt/planar cell polarity (PCP) and the Wnt/Ca^2+^ pathway, which coexist and share certain overlapping components with the canonical one [44]. The Wnt/PCP pathway is also known as the Wnt/JNK pathway. Upon triggering, it cooperates with receptor tyrosine kinase-like orphan receptor 2 (ROR2)/ receptor-like tyrosine kinase (RYK) and activates small GTPases to control cell polarity, cellular cytoskeletal arrangements, adhesion and migration [35, 41, 44]. The Wnt/Ca^2+^ pathway activates dishevelled (DVL) and phospholipase C (PLC), causing the release of intracellular Ca^2+^ ions, which in turn activates protein kinase C (PKC), phosphatase calcineurin and calcium calmodulin mediated kinase II (CaMKII) that drive transcription of nuclear factor of activated T cells (NFAT)/AP1 gene [35].

### Wnt signaling in osteosarcoma

In recent years, the profound implication of Wnt cascade in human cancer development has been well established [37]. Dysregulation in Wnt signaling pathway predisposes patients to multiple cancers. It has been reported that nearly 80% of the colorectal cancer patients harboring mutations in APC and β-catenin genes [44]. The Wnt receptor FZD6 was reported to be frequently amplified in breast cancer, with a particular increased incidence in triple-negative breast cancer [45]. Meanwhile, genomic analysis also revealed 46% of gastric cancer harboring deregulation of Wnt cascade [46].

In addition, deficiency or hyperactivation of Wnt may be responsible for the cancer aggressive behavior or therapy response. In glioblastoma, the Wnt signal is closely correlated with sensitivity to chemo- and radio-therapy [47, 48]. Another study showed that the Wnt co-receptor ROR1 is expressed in chronic lymphocytic leukemia cells, allowing for targeting these cells with specific monoclonal antibody (cirmtuzumab) [49, 50]. Besides, Wnt pathway could participate in angiogenesis [39, 43] and immune-surveillance, which are key events in tumor cell dissemination and metastasis [6]. It is well-known that Wnt/β-catenin pathway plays a crucial role in bone tumor microenvironment. Besides the implication of the canonical Wnt/β-catenin signaling in angiogenesis [51–54] and immune evasion [55–57], it balances bone remodeling by favoring bone formation but repressing bone resorption [54, 58, 59]. Hyper-activation of Wnt would disrupt this balance in favor of osteosarcoma development and metastasis.

In similar, alteration of the constitutive components of Wnt cascade is also considered as one of the major molecular mechanisms in osteosarcoma initiation and progression [60]. These alterations comprise mutations, amplifications, deletion, promoter hypermethylations, and changes in subcellular localization [61]. A variety of Wnt components, ligands, receptors/co-receptors, and antagonists are dysregulated in osteosarcoma, implying an prominent role of Wnt cascade in tumorigenicity and metastatic dissemination [62]. For example, expression of β-catenin and its nuclear effector-LEF1 are found both elevated in osteosarcoma tissues [63], and osteosarcoma cells than that in corresponding control [64, 65], respectively. LRP5 was frequently expressed in osteosarcoma, and correlated significantly with the chondroblastic subtype of osteosarcoma and metastasis [66–68]. Likewise, another in vivo study also found that dominant negative LRP5 impeded the osteosarcoma tumorigenic potential and metastasis [69]. Meanwhile, secreted Fzd-related protein 2 (sFRP2) was highly expressed in osteosarcoma patients and inversely correlated with survival. sFRP2 overexpression could induce angiogenesis and drive osteoblast precursors into osteosarcoma phenotype [39, 70], while knockdown of sFRP2 impaired its metastatic and invasive behavior [71]. Conversely, inactivation of Wnt inhibitory factor 1 (Wif-1) has been closely correlated with radiation-induced osteosarcoma [72]. Overexpression of another Wnt antagonist, Dickkopf-3 (DKK-3), also retarded tumor growth and pulmonary metastasis in xenograft model [73]. Taken together, these findings highlight the diverse role of Wnt signaling in osteosarcoma.

## Regulatory network of lncRNAs and Wnt signaling pathway in osteosarcoma

### Wnt-related LncRNAs signaling in cell proliferation of osteosarcoma

Sustained and uncontrolled cell proliferation is an important defining hallmark of carcinogenesis [74]. The relationship between Wnt and osteosarcoma cell proliferation is already well evident [72]. Recently, enormous studies have clarified the involvement of Wnt-related lncRNAs in controlling cell proliferation of osteosarcoma as well.

By analyzing the lncRNA expression pattern in different gene expression omnibus (GEO) datasets, Yao Q et al. observed that LINC01128 was upregulated in osteosarcoma and negatively correlated with overall survival. In vitro and in vivo study both confirmed that LINC01128 facilitated cell proliferation. Mechanistic exploration revealed that LINC01128 regulated MMP2 through sponging miR-299-3p and activation of Wnt/β-catenin signaling pathway in osteosarcoma [75].

Lymphoid enhancer-binding factor 1 antisense RNA 1 (LEF1-AS1) was firstly identified to be remarkably correlated with overall and recurrence-free survival of colorectal cancer patients via RNA-seq and microarray data from The Cancer Genome Atlas (TCGA) and GEO [76]. One investigation showed that LEF1-AS1 is upregulated in osteosarcoma with capability to enhance cell proliferation and to stimulate Wnt pathway by sponging heterogeneous nuclear ribonucleoprotein L (HNRNPL) to stabilize mRNA of LEF1. Moreover, the effect of LEF1-AS1 deletion could be partially rescued by LEF1 overexpression, which further confirmed a regulatory loop of LEF1-AS1/HNRNPL/LEF1-AS1 in osteosarcoma [64].

Similarly, another recent study showed that small nucleolar RNA host gene 10 (SNHG10) was markedly overexpressed in osteosarcoma compared with adjacent healthy counterparts by quantitative real time polymerase chain reaction (qRT-PCR) and fluorescence in situ hybridization (FISH) analysis [77]. Consistently, higher expression level of SNHG10 is also observed in osteosarcoma cell lines, including MG-63, SW-1353, U-2OS, Sasos-2, HOS, and 143B, when compared with osteoblastic cell line hFOB1.19. Colony formation and CCK-8 assay showed that SNHG10 silencing attenuated osteosarcoma cell proliferation in vitro. Moreover, SNHG10 promoted osteosarcoma tumorigenesis in xenograft tumor model. Luciferase reporter assay and RIP further revealed that SNHG10 acted as ceRNA to sponge miR-182-5p and increase expression of FZD3. Accordingly, the Wnt cascade was stimulated, leading to in-nuclear accumulation of β-catenin and increased expression of target genes including Cyclin D1, cluster of differentiation 44 (CD44), TCF-1 and Axin-2 [77].

### Wnt-related LncRNAs in regulation of cellular apoptosis

Wnt signaling has been well functionally identified to govern cell apoptotic death in osteosarcoma for years [78, 79]. The Wnt signaling-related lncRNAs have been shown to exert important roles in regulation of cell apoptosis in recent years.

LncRNA down syndrome cell adhesion molecule antisense RNA 1 (DSCAM-AS1) was originally identified as the most abundant Apo-ERα-regulated lncRNA (AER-lncRNA) in breast cancer [80]. Subsequent work ascribed an oncogenic role for DSCAM-AS1, with the expression in colorectal cancer associated with metastasis, advanced stage and poor overall survival [81], and in breast cancer linked with tumor progression and tamoxifen resistance [82, 83]. Similarly, DSCAM-AS1 was proven highly expressed in osteosarcoma cell lines. In vitro experiments have shown that DSCAM-AS1 depletion could dramatically enhance cell apoptosis in osteosarcoma and inactivate Wnt signaling pathway [84].

Lin H and colleagues identified another highly expressed lncRNA lnc-MAP6-1:3 in osteosarcoma. Functional experiments convinced that lnc-MAP6-1:3 could accelerate cell proliferation, colony formation and inhibit cell apoptotic death via regulating Bax/Bcl-2 and Wnt/β-catenin pathway [85].

TMPO antisense RNA 1 (TMPO-AS1) has been reported to be overexpressed, whereas miR-199a-5p was downregulated in both osteosarcoma tissues and cell lines. Knockdown of TMPO-AS1 repressed cell proliferation, promoted apoptosis and restrained Wnt/β-catenin by directly sponging miR-199a-5p to regulate WNT7B. In addition, the suppression by miR-199-5p inhibitor on osteosarcoma could be rescued by WNT7B knockdown, while this effect could be further abolished by LiCl treatment (activator of Wnt pathway). Taken together, these studies suggested an essential role of TMPO-AS1/miR-199a-5p/WNT7B axis in osteosarcoma tumorigenesis [86].

CAT104 is established as an oncogene in several cancers including gastric cancer [87], leukemia [88], and osteosarcoma [89]. CAT104 was shown to be upregulated in osteosarcoma MG-63 and OS-732 cell lines, and its knockdown could restrain cell proliferation and promote apoptosis. Furthermore, CAT104 was found to regulate miR-381 to target zinc finger E-box-binding homeobox 1 (ZEB1) as well as Wnt/β-catenin pathways to exert its oncogenic effects on osteosarcoma cells [89].

### LncRNA in regulation of Wnt-dependent metastasis and invasion of osteosarcom*a*

Early aggressive metastasis could contribute to rapid progression and unfavorable prognosis of osteosarcoma [90]. There are unequivocal evidences that Wnt cascade-related lncRNAs play a crucial role in regulating invasion, migration and metastasis of osteosarcoma.

Maternally expressed gene 3 (MEG3) is located on human chromosome 14q32.3, and defined as a tumor suppressor in several human cancers [91], such as nasopharyngeal carcinoma [92], breast cancer [93, 94] and ovarian cancer [95]. A recent study reported that MEG3 was aberrantly expressed in osteosarcoma. Forced expression of MEG3 hindered osteosarcoma cell proliferation and migration in vitro, and retarded tumor growth in vivo. Further assay showed that MEG3 negatively regulated miR-184 and downstream effector of Wnt signaling pathway, such as β-catenin, TCF4 and c-MYC. Moreover, the inhibitory effect of MEG3 could be reversed by miR-184 mimic, suggesting a cooperative regulation of MEG3 and miR-184 in osteosarcoma [96].

Another study showed that urothelial carcinoma associated 1 (UCA1) could enhance cell viability, migration and invasion [97]. Expression level of chemokine receptor- C-X-C motif chemokine receptor 4 (CXCR4) has been well-documented to be strongly associated with osteosarcoma invasion and metastasis [98, 99]. Meanwhile, miR-301a was also shown to have a cancerous function in several human cancers including osteosarcoma [100]. Its expression is correlated with cell migration and doxorubicin resistance in osteosarcoma cell lines [101, 102]. Of note, the expression of UCA1 expression was positively related with CXCR4 and miR-301a, and UCA1 could upregulate miR-301a and subsequently increase CXCR4 expression. Moreover, the inhibitory effect of UCA1 knockdown in osteosarcoma cells could be blocked by overexpression of miR-301a, but reversed by CXCR4 inhibition. Interestingly, UCA1 could activate Wnt/β-catenin signaling pathway and nuclear factor kappa-B (NF-κB) via regulation of miR-301a/CXCR4 axis [97].

Tian Z et al. demonstrated that lncSox4 is upregulated in osteosarcoma cell lines and tissues, and boosted cell migration by stabilizing β-catenin expression. Intriguingly, Wnt agonist CID11210285 abrogated the inhibitory effect on MG-63 cells induced by lncSox4 knockdown, while Wnt inhibitor IWP-3 reversed the oncogenic effect on MG-63 caused by lncSox4 overexpression [103]. Besides, lncRNA CAMK2D-associated transcript 1 (C2dat1) knockdown mitigated osteosarcoma cell invasion and migration by regulating miR-34a-5p/Sirt1 network and Wnt signaling pathway [104].

Enforced (epithelial-mesenchymal transition) EMT is a process in which epithelial cells are transitioned into mesenchymal phenotype, resulting in promoted invasive capacities and unsatisfactory survival rate of cancer patients [105]. EMT can be characterized by increased mesenchymal markers including N-cadherin, Slug, Twist, Vimentin and Fibronectin, but decreased epithelial markers such as E-cadherin [6]. Gastric carcinoma proliferation enhancing transcript 1 (GHET1) is an upregulated lncRNA in osteosarcoma cell lines in comparison with normal osteoblastic cells. GHET1 knockdown was reported to inhibit osteosarcoma cell migration, invasion and EMT, at least in part via regulation of Wnt/β-catenin pathway [106].

LncRNA CRNDE was found highly expressed in both osteosarcoma tissues and cell lines. In line with the clinical finding, in vitro study showed that ablation of CRNDE restrained invasion of osteosarcoma cells, downregulated N-cadherin, vimentin and snail, while upregulated expression of E-cadherin and ZO-1. Mechanistically, CRNDE could enhance GSK-3β phosphorylation to trigger Wnt/β-catenin signaling pathway [107].

Taurine upregulated gene 1 (TUG1) was also significantly overexpressed in osteosarcoma tissues. Its expression level was positively correlated with distant metastasis and further indicated poor overall and recurrence-free survival [90]. Further study confirmed that enhancer of zeste homolog 2 (TUG1) regulated cell metastatic dissemination by mediating hypoxia-inducible factor -1alpha (HIF-1α) via miR-143-5p [90] or EZH2 via miR-144-3p [108]. In addition, inhibition of TUG1 inactivated Wnt/β-catenin pathway, and LiCl could partly abolish the inhibitory effect on cell migration and EMT process induced by TUG1 knockdown [108]. Consistently, another study by Yang GH et al. [109] showed that miR-425-5p overexpression could inhibit osteosarcoma invasion and migration through directly binding to metastasis-associated lung adenocarcinoma transcript 1 (MALAT1) and TUG1, and blocked their activation of Wnt pathway both in vitro and in vivo [109]. Collectively, inhibition of TUG1 and subsequent inactivation of Wnt may be a promising strategy in treating osteosarcoma.

### Wnt-related LncRNAs in regulation of cell cycle

The cell cycle is coordinated by dynamics of master cyclins and cyclin-dependent kinases (CDKs) complex to regulate the sequence and timing of proliferation events [110]. Disruption of cell cycle progression is an established hallmark of cancer, which may result in uncontrolled cellular proliferation [111]. Phosphorylation of β-catenin is able to trigger cyclin production, which links the Wnt signaling to cell cycle regulation [110, 112]. Given the pivotal role of Wnt/β-catenin in osteosarcoma cell cycle modulation, some studies have sought to identify the corresponding associated lncRNAs.

High BE503655 expression has been observed in osteosarcoma tissues compared with controls, which is also inversely closed related to Enneking stage, histological grade and distant metastasis [63]. Meanwhile, BE503655 is also highly expressed in osteosarcoma MG-63 and HOS cell lines. Flow cytometry detection revealed that BE503655 silencing obviously arrested the osteosarcoma cells in G0 and G1 phase, while obstructed S phase entry. Meanwhile, expression of BE503655 was positively associated with β-catenin. BE503655 knockdown in osteosarcoma HOS cells could downregulate β-catenin as well as an array of Wnt cascade downstream targets, including c-Myc (a proto-oncogene), Cyclin D and MMP2 [63]. Moreover, the cell cycle arrest effect induced by BE503655 knockdown was blocked by β-catenin overexpression, thus corroborating BE503655 functions in a Wnt/β-catenin dependent manner in osteosarcoma [63].

Located at the chromosomal locus 7p15.2, lncRNA HOXA transcript at the distal tip (HOTTIP) has been found to be frequently abnormally expressed in various cancer types, containing gastric cancer [113, 114], lung adenocarcinoma [115], head and neck squamous cell carcinoma [116], pancreatic cancer [117, 118]. Importantly, HOTTIP was the first lncRNA documented to regulate Wnt expression in osteosarcoma. Li Z et al. [119] showed that HOTTIP was overexpressed in both osteosarcoma tissues and cell lines. In vitro experiments revealed that enhanced HOTTIP expression markedly promoted MG-63 osteosarcoma cells into S phase, while HOTTIP downregulation significantly arrested the cell cycle in G1 phase. Mechanistically, HOTTIP overexpression or knockdown in MG-63 cell caused parallel changes in β-catenin expression, implying that the β-catenin was directly regulated by HOTTIP. Moreover, HOTTIP could increase expression of cell cycle-related proteins (Cyclin D1 and CDK4) dependent on Wnt/β-catenin pathway [119].

### Wnt-related LncRNAs in osteosarcoma stemness

Cancer stem cells (CSCs), also known as tumor initiating cells (TICs), are a small functional subpopulation of cells that exhibit stem-like gene expression [120, 121]. They are considered to be mainly responsible for maintaining tumor cell vitality through self-renewal and infinite proliferation abilities, especially under hypoxia conditions [21, 122]. Besides, CSCs are more resistant to conventional chemotherapy/radiotherapy and even latest immunotherapy [120], which may contribute toward cancer treatment failure and worsening of patients’ prognosis [123, 124].

Wnt signaling has been well documented to maintain stem cells in a pluripotent state [35]. Meanwhile, a growing number of studies confirmed a determinant role of lncRNAs in sustaining stemness of CSCs based on analysis of transcriptome sequencing [120]. In one investigation, Li F et al. reported that lncRNA HOXA transcript antisense RNA, myeloid-specific 1 (HotairM1) is downregulated in CSCs of colorectal carcinoma and uveal melanoma [125]. Further mechanical assay showed that HotairM1 could recruit EZH2 and suppressor of zeste 12 (SUZ12), and consequently form a reciprocal regulation loop with HOXA1-Nanog to augment the tumor stemness effect [125]. More recently, another study reported that lncRNA LHFPL3-AS1 may inhibit apoptosis and maintain stemness viability of melanoma CSCs by sequestration of miR-181a-5p to upregulate Bcl-2 expression [126].

In osteosarcoma, lncRNAs distal-less homeobox 6 antisense 1 (DLX6-AS1) was highly expressed in osteosarcoma tissue and cell lines [123, 127, 128]. Moreover, its expression was significantly correlated with advanced TNM stage, tumor grade, distant metastasis and poor prognosis [123, 127]. Mechanistically, DLX6-AS1 could function as a ceRNA to interact with miR-129-5p to target delta-like homologue 1 (DLK1), and thus form a reciprocal feedback loop to activate Wnt cascade, thereby promoting stemness of osteosarcoma [123]. Taken together, these studies exemplify that specifically targeting Wnt signaling-associated lncRNAs may help promote CSCs elimination, prevent osteosarcoma recurrence, and thus paving a new pathway in the treatment.

### Wnt-related LncRNAs in osteosarcoma drug resistance

Drug resistance remains a multifaceted obstacle in cancer treatment, which may be attributed to tumor heterogeneity, survival pathway activation, and cytotoxic drug efflux [40]. Osteosarcoma patients often develop drug resistance, which may in turn largely impairs the therapeutic effect of chemotherapy, and eventually leads to tumor recurrence [124, 129]. It is necessary to undermine the mechanisms in osteosarcoma chemoresistance, and thus to provide innovative strategy for efficacy improvement in chemotherapy.

Deregulated Wnt pathway has been demonstrated to favor resistance to conventional chemotherapy [57]. Recently, emerging studies have also highlighted a pivotal role of Wnt signaling-related lncRNA in regulation of chemotherapy sensitivity [116]. For example, HNF1A-AS1 level was found to be obviously upregulated in cisplatin-resistant cervical cancer cell line. Exosomes carrying HNF1A antisense RNA 1 (HNF1A-AS1) enhanced drug resistance by sponging miR-34b to elevate tuftelin1 (TUFT1) expression [130]. P-glycoprotein (P-gp/ABCB1), encoding by multidrug resistance 1 (MDR1) gene, is regulated by β-catenin/TCF/LEF-binding sites [41] and defined as a key mediator in acquired chemoresistance [131]. In breast cancer, overexpression of lncRNA growth arrest-specific 5 (GAS5) could remarkably enhance adriamycin sensitivity, while suppress drug efflux and ABCB1 expression via regulation of miR-221-3p/DKK2 axis and Wnt/β-catenin pathway [132].

Platinum-based chemotherapy, mainly cisplatin, has been widely applied to suppress osteosarcoma growth and metastasis [133]. Resistance to cisplatin is often associated with poor prognosis in osteosarcoma patients [134]. Recently, an investigation showed that HOTTIP could regulate the cisplatin sensitivity in osteosarcoma [119]. In vitro assay showed that HOTTIP expression conferred cisplatin resistance through activation of Wnt/β-catenin signaling pathway. Upon treatment of Wnt/β-catenin inhibitor, the cellular resistance to cisplatin could be reversed. Thus, HOTTIP was proposed to sensitize osteosarcoma cells in cisplatin-based chemotherapy via Wnt pathway, uncovering a novel network in osteosarcoma treatment [119].

## Possible involvement of lncRNA in mutation of Wnt signaling

Mutation-induced activation of Wnt pathway frequently drives tumorigenesis and therapy resistance. The excessive activity of Wnt/β-catenin pathway could be achieved via following unwanted mutations. Firstly, inactivation of the β-catenin destruction complex initiated by inactivating mutations of APC, AXIN1 and AXIN2 or activating mutations in β-catenin, is postulated to drive WNT-independent growth [135–137]. Secondly, loss of RNF43 and ZNRF3 could sustain an over-abundance status of cell surface WNT receptors, which is assumed to promote WNT-dependent tumor growth [138–140]. There are rare findings regarding such function of lncRNA in cancer research. LncRNA CA7-4 decoys MIR877-3p, the latter of which triggers the reduction of CTNNBIP1 (catenin beta interactin protein 1) by interacting with its 3’UTR and the upregulating CTNNB1 [141]. Thus, the CTNNB1-encoded β-catenin might acquire amplified activity, which lacks direct evidence. Future investigations regarding the role of lncRNA in mutation-driven Wnt signaling alterations in tumorigenesis will benefit therapeutic decisions.

## The clinical significance of lncRNAs involved in Wnt pathway in osteosarcoma

Given the fact that most lncRNAs are tissue- or cancer-context-specific, stable in circulatory form [142], and easy for detection [143, 144], it is possible that lncRNAs may serve as ideal diagnostic and prognostic biomarkers, as well as promising therapeutic candidates [27].

Abnormal expression of multiple Wnt-related lncRNAs has been demonstrated to be closely associated with clinicopathological features of osteosarcoma. For instance, FLVCR1 antisense RNA 1 (FLVCR1-AS1) is highly expressed in osteosarcoma than adjacent normal tissue, and its expression is positively associated with tumor size, WHO grade and distant metastasis in osteosarcoma patients. Moreover, patients with FLVCR1-AS1 upregulation have unfavorable survival rate [145], indicating that FLVCR1-AS1 may be viable biomarker in osteosarcoma.

LncRNA small nucleolar RNA host gene 1 (SNHG1), one of dysregulated lncRNAs in multiple cancer, is shown to be also involved in pathology of osteosarcoma. SNHG1 is highly expressed in osteosarcoma and positively correlated with tumor size, TNM stage and lymph node metastasis [146].

LncRNA AWPPH expression was elevated in osteosarcoma tissues compared with paracancerous controls [147]. Overexpression of AWPPH was significantly correlated with advanced tumor stage, tumor size, metastasis, and conferred reduced overall survival rate [148].

Expression of HNF1A-AS1 was significantly increased in osteosarcoma tissues in contrast to adjacent normal tissue. Upregulated HNF1A-AS1 overexpression was significantly associated with advanced clinical stage, distant metastasis [149]. Multivariate Cox proportional hazards analysis suggested that HNF1A-AS1 was an independent risk factor of overall survival in osteosarcoma patients [150]. Moreover, serum HNF1A-AS1 was capable to separate osteosarcoma patients from healthy counterparts with the area under curve (AUC) of 0.849 in receiver operating characteristic (ROC) curve [150].

The expression levels of long stress-induced noncoding transcript 5 (LSINCT5) were upregulated in osteosarcoma tissues and cell lines. High LSINCT5 level was positively correlated with malignant clinicopathological features, including advanced Enneking stage and histological grade, larger tumor size, and distant metastasis [151]. Besides, osteosarcoma patients with high expression of LSINCT5 showed a trend toward decreased overall survival [151, 152].

Dai J et al. reported that ITGB2 antisense RNA1 (ITGB2-AS1) was upregulated in osteosarcoma tissues, and were negatively correlated with prognosis of osteosarcoma patients [153]. LncRNA actin filament-associated protein 1-antisense RNA 1 (AFAP1-AS1) has been validated to be highly expressed in osteosarcoma tissues than that of adjacent tissues [154–156], and was negatively correlated with prognosis of osteosarcoma patients [157]. Collectively, the Wnt-related lncRNAs may be potential predictors for clinical outcomes in osteosarcoma.

## Potential of Wnt-related lncRNAs as biomarkers and therapeutic targets in osteosarcoma

Exosomes are small membranous vesicles, which transport cargoes of protein and genetic materials in biological fluids. Accumulating evidence support lncRNAs are enriched in exosomes from osteosarcoma model [158, 159]. Liquid biopsy has been developed to detect early development, advanced and metastasis of osteosarcoma by profiling circulating tumor DNA (ctDNA) and miRNA, but less focus on either exosomes or lncRNA (Using Liquid Biopsy in the Treatment of Patient with OS). Future tumor data profile trends to include lncRNAs, especially exosomal lncRNAs for diagnostic and prognostic purposes.

Above, we have detailed illustrated functions of various Wnt-related lncRNAs in tumor growth, migration, invasion and drug resistance. Wnt-related lncRNAs, including MEG3, MALAT1, TUG1, HOTTIP, GAS5, CRNDE, UCA1 and LSINCT5 have been detected in secreted exosomes, suggesting exosomal lncRNAs can be developed into convenient and noninvasive biomarkers for diagnosis and prognosis [160–166]. However, to our knowledge, the potential of using exosomal lncRNAs in osteosarcoma samples or within body fluids as a source for biomarkers has not been reported. Besides, large gaps still remain on therapeutic application of lncRNAs in osteosarcoma. Firstly, technical problems, such as extraction of exosomes from different specimens must be solved. Sensitivity and specificity of certain exosomal lncRNA as biomarkers could be clarified once exosomes yields are prepared. Secondly, identification of derivation of certain exosomal lncRNAs is necessary for diagnostic and therapeutic purpose. For example, non-tumor cell, especially stem cells-derived lncRNAs can activate diverse target cell activities in modeling tumor microenvironment. Thirdly, delivering lncRNA modulation systems to target live cells remains challenging. Traditional viral vectors carry with high immunogenicity and toxicity. In converse, non-viral vectors are recognized as an alternative to drastic immune response caused by viral vectors. Exosomes are under development of delivery system. More attractively, nucleic-acid aptamers have been demonstrated as another promising target delivery method via specific cell surface receptors, which possessed higher cell-type specific and gene-editing effect [167].

Currently, there are three novel therapeutic strategies focusing on lncRNAs in osteosarcoma, including small interfering RNA (siRNA), an antisense oligonucleotide (ASO)-based strategies and molecular inhibitors, all of which modulate lncRNA expression by gene editing [168]. RNAi is an effective method to reduce the amount or activity of target lncRNA via several mechanisms. One is designing siRNA as complementary sequence to lncRNA in purpose to promote lncRNA binding and subsequent degradation, which is efficient for cytoplasmic lncRNAs since the process of siRNA-lncRNA interaction exists predominantly in the cytoplasm. Another mechanism is to target lncRNA-protein interaction. RNAi molecule could compete with the protein for the binding site or disrupting the binding site of protein when interacting with target lncRNA. Conversely, ASOs strategy shows more advantage over RNAi in terms of targeting both nuclear lncRNAs. ASOs bind to target lncRNA to induce gene silencing [169]. ASOs developed to target MALAT1 have shown promising results in cancer treatment [170]. However, both ASO and RNAi strategies have shortcomings in terms of non-specific targets and transient modulation [171–173]. Several improvements have been made to solve the problem of target specificity. One striking methodology is CRISPR/Cas9. The Cas9 nuclease can guide site-specific DNA cleavage or deletion of lncRNA promoters by an optimized single-guide RNA (sgRNA). It has been applied in cancer research especially as potent lncRNA therapeutics with the development of aptamerliposome-CRISPR/Cas9 chimera. One chimera has been adapted to specifically bind to the prostate membrane antigen on prostate cancer cells [174]. The limitations of the preclinical application of CRISPR/Cas9 are off-target effects and limited lncRNAs as targets. Another very-recently developed technique is CRISPR/Cas13 system, which adopts cas13 endoribonuclease (also known as C2c2) to manipulate reverse genetic edit on target RNA [175]. This promising approach has demonstrated biological relevance of lncRNAs in cancer therapeutics mechanically, which requires further exploration in osteosarcoma.

## Conclusions and future perspectives

Osteosarcoma is a highly aggressive tumor with propensity for local invasion, relapse as well as distant metastasis [176–179]. A comprehensive understanding of the underlying mechanisms involved in osteosarcoma pathogenesis is urgently needed to advance the new therapies for osteosarcoma patients [72].

**Fig. 3:**
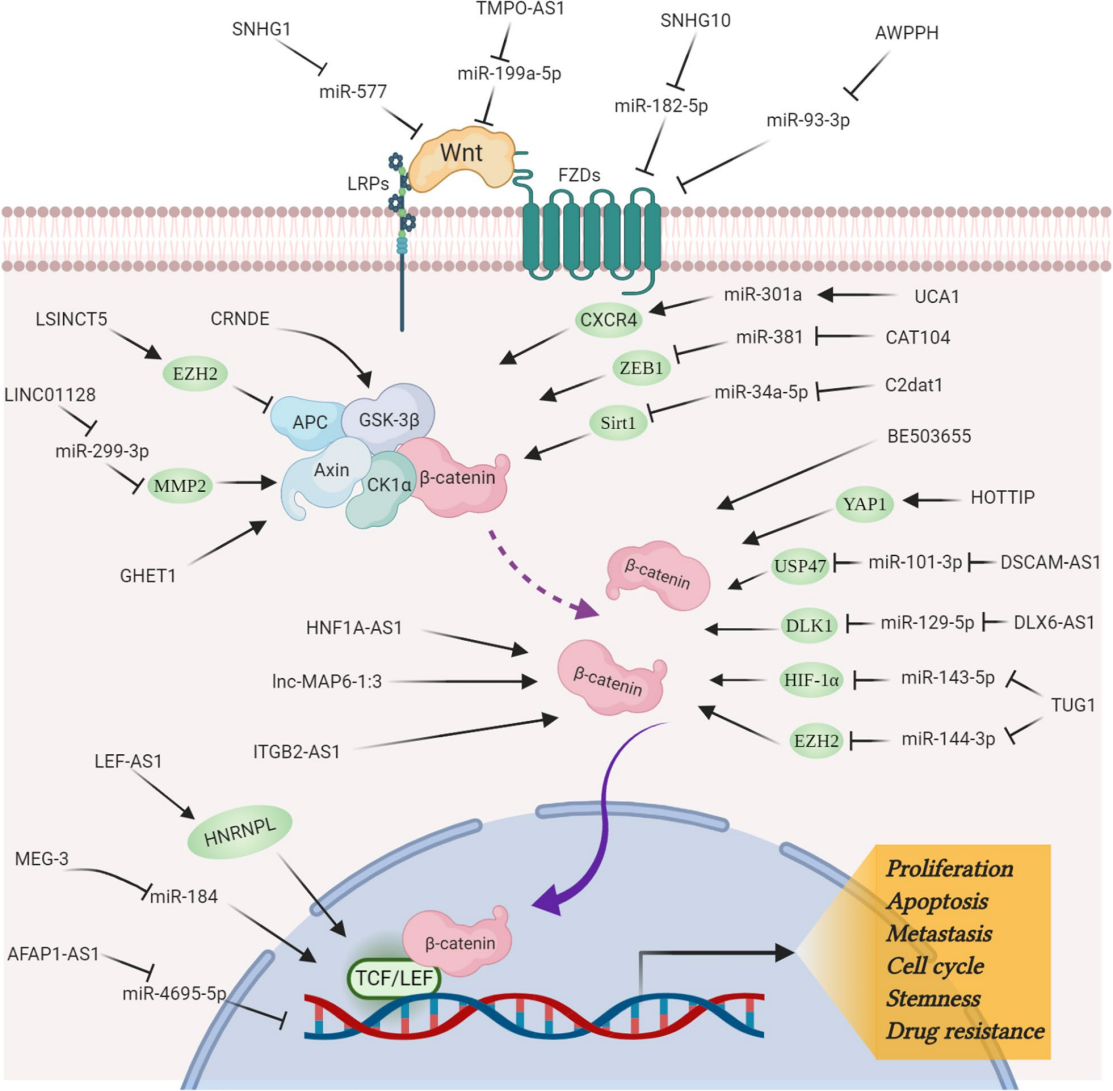
Emerging interplays between lncRNAs and Wnt signaling pathway. The schematic drawing shows the regulatory network of oncogenic and tumor suppressor lncRNAs in Wnt cascades to affect the expression of downstream target genes, which consequently contributes to the pathogenesis of osteosarcoma. Figure created with BioRender.com

The Wnt pathway is a highly conserved and versatile pathway that plays a central role in governing cellular proliferation, apoptosis, stemness, drug resistance, and other crucial hallmarks implicated in pathophysiology of cancer [180, 181]. In the light of these premises, Wnt signaling cascades are defined as interesting and promising therapeutic targets for osteosarcoma treatment [182]. However, owing to its complex nature, the development of drug targeting Wnt signaling may lead to inevitable side effects, and thus has been considered to be impossible for a long time [35]. In recent years, Wnt-targeting therapeutics in other cancer types are in different phases of pre-clinical or early clinical trials, while those for osteosarcoma are still scarcely observed [6, 41]. Recently, lncRNAs are gaining researchers’ attention as an alternative strategy for cancer treatment [22, 183, 184]. The increasing insights into the crosstalk between lncRNAs and Wnt in osteosarcoma may lead to the development of promising pharmacological candidates (Shown in Fig. 3; Table 1). Due to its tissue-, cell- and even time-specific expression context, Wnt-related lncRNAs may provide us with a more refined strategy, and less adverse effects than currently available treatment options in osteosarcoma.

**Table 1:** Overview of Wnt signaling pathway-related lncRNAs in development of osteosarcoma

LncRNA	Expression pattern	Interaction with Wnt pathway	Target genes	Associated clinical values	Cellular physiological functions	Regulatory modality	Refs.
AFAP1-AS1	↑	Activating Wnt/β-catenin	miR-4695-5p/TCF4	Prognosis	Proliferation, invasion	Transcriptional	[157]
AWPPH	↑	Activating Wnt/β-catenin	miR-93-3p/FZD7	Advanced stage, tumor size, metastasis, survival	Proliferation, migration, invasion	Transcriptional	[148]
BE503655	↑	Activating Wnt/β-catenin	-	Enneking stage, DM, histological grade	Proliferation, cell cycle, invasion, migration	Unknown	[63]
C2dat1	↑	Activating Wnt/β-catenin, p38/ERK/AKT	miR-34a-5p/Sirt1	-	Cell viability, migration, invasion, apoptosis	Transcriptional	[104]
CAT104	↑	Activating Wnt/β-catenin, JNK	miR-381/ZEB1	-	Proliferation, migration, invasion, apoptosis	Transcriptional	[89]
CRNDE	↑	Activating Wnt/β-catenin	GSK-3β	-	Proliferation, invasion, apoptosis, cell cycle arrest, EMT	Post-transcriptional	[107]
DLX6-AS1	↑	Activating Wnt	miR-129-5p/DLK1	Prognosis	Stemness	Transcriptional	[123]
DSCAM-AS1	↑	Activating Wnt/β-catenin and AKT/mTOR	miR-101-3p/USP47	-	Proliferation, migration, invasion, apoptosis	Transcriptional	[84]
FLVCR1-AS1	↑	Activating Wnt/β-catenin	CTNNB1, SOX4, CCND1, CCND2, Myc	Tumor size, WHO grade, DM, survival	Proliferation, migration and invasion	Unknown	[145]
GHET1	↑	Activating Wnt/β-catenin	-	-	Proliferation, migration, invasion, EMT, apoptosis	Unknown	[106]
HNF1A-AS1	↑	Activating Wnt/β-catenin	-	Clinical stage, DM, overall survival	Proliferation, metastasis	Unknown	[149]
HOTTIP	↑	Activating Wnt/β-catenin	-	Chemoresistance	Proliferation, cell cycle, cisplatin resistance	Unknown	[119]
ITGB2-AS1	↑	Activating Wnt/β-catenin	-	Prognosis	Proliferation, apoptosis, migration, invasion	Unknown	[153]
LEF1-AS1	↑	Activating Wnt	HNRNPL/LEF1	-	Proliferation, migration, invasion	Transcriptional	[64]
LINC01128	↑	Activating Wnt/β-catenin	miR-299-3p/MMP2	-	Proliferation, migration, and invasion	Transcriptional	[75]
Lnc-MAP6-1:3	↑	Activating Wnt/β-catenin and Bax/Bcl-2	-	-	Proliferation, apoptosis	Post-transcriptional	[85]
LncSox4	↑	Activating β-catenin	-	-	Viability, migration	Post-transcriptional	[103]
LSINCT5	↑	Activating β-catenin	EZH2/APC	Prognosis	Proliferation, tumor growth	Transcriptional	[152]
MEG3	↓	Inhibit Wnt/β-catenin	miR-184/TCF4 and c-Myc	-	Cell viability, metastasis, apoptosis	Transcriptional	[187]
TMPO-AS1	↑	Activating Wnt/β-catenin	miR-199a-5p/WNT7B	-	Proliferation, apoptosis	Transcriptional	[86]
SNHG1	↑	Activating Wnt/β-catenin	miR-577/WNT2B	Tumor size, TNM stage, LNM	Proliferation, migration, EMT	Transcriptional	[146]
SNHG10	↑	Activating Wnt/β-catenin	miR-182-5p/FZD3	-	Proliferation, invasion	Transcriptional	[77]
TUG1	↑	Activating Wnt/β-catenin	miR-144-3p/EZH2	-	Proliferation, migration, invasion, apoptosis	Transcriptional	[108, 109]
UCA1	↑	Activating Wnt/β-catenin and NF-κB	miR-301a/CXCR4	-	Cell viability, migration, invasion, apoptosis	Transcriptional	[97, 188]

*AFAP1-AS1* actin filament-associated protein 1-antisense RNA 1, *APC* adenomatous polyposis coli, *C2dat1* CAMK2D-associated transcript 1, *CXCR4* C-X-C motif chemokine receptor 4, *DLK1* delta-like homologue 1, *DLX6-AS1* distal-less homeobox 6 antisense 1, *DM* distant metastasis, *DSCAM-AS1* down syndrome cell adhesion molecule antisense RNA 1, *EMT* epithelial-mesenchymal transition, *EZH2* enhancer of zeste homolog 2, *FLVCR1-AS1* FLVCR1 antisense RNA 1, *FZD3* Frizzled 3, *TCF4* transcriptional factors T cell factor4, *GHET1* Gastric carcinoma proliferation enhancing transcript 1, *GSK-3β* glycogen synthase kinase 3β, *HNF1A-AS1* HNF1A-antisense 1, *HNRNPL* heterogeneous nuclear ribonucleoprotein L, *HOTTIP* HOXA transcript at the distal tip, *ITGB2-AS1* ITGB2 antisense RNA1, *LEF1* lymphoid enhance factor 1, *LEF1-AS1* Lymphoid enhancer-binding factor 1 antisense RNA 1, *LNM* lymph node metastasis, *LSINCT5* long stress-induced noncoding transcript 5, *MEG3* maternally expressed gene 3, *MMP2* maxtrix metalloproteinase 2, *Sirt1* Sirtuin 1, *SNHG1* small nucleolar RNA host gene 1, *SNHG10* small nucleolar RNA host gene 10, *TMPO-AS1* TMPO antisense RNA 1, *TUG1* taurine upregulated gene 1, *UCA1* urothelial carcinoma associated 1, *USP47* ubiquitin-specific peptidase 47, *ZEB1* zinc finger E-box-binding homeobox 1, **↑**:upregulated, **↓**:downregulated

Considering the indispensable and diverse role played by lncRNAs in carcinogenesis, it is not surprising that Wnt-related lncRNAs are extensively involved in osteosarcoma progression. It is worthy of note that the majority of currently studies are focused on the upstream regulatory lncRNAs, while the downstream lncRNA targets of Wnt cascade remains largely unexplored. Hence, future studies are warranted to also elucidate the respective role as downstream lncRNA targets. Besides, other alternative regulatory networks concerning the role of Wnt-related lncRNAs in osteosarcoma, in addition to ceRNA, should be addressed. Moreover, it is of great necessity to further validate the sensitivity and specificity of lncRNAs as biomarkers in clinical settings. Established serum markers, such as alkaline phosphatase (ALP) and lactate dehydrogenase (LDH), have been widely adopted to aid in diagnosing osteosarcoma for decades [185, 186], and whether these Wnt-related lncRNAs have more advantages over these markers need to be further verified. Due to the easy accessibility and routine availability, the Wnt signaling-related lncRNAs with excellent diagnostic performance may be promising candidates in near future. A more comprehensive understanding of the role of Wnt signaling-related lncRNAs may eventually rationalize novel, yet unexplored, therapeutic opportunities for individualized treatment in osteosarcoma.

## Data Availability

Not applicable.

## Abbreviations

AER-lncRNA: Apo-ERα-regulated lncRNA
AFAP1-AS1: Actin filament-associated protein 1-antisense RNA 1
ALP: Alkaline phosphatase
APC: Adenomatous polyposis coli
ASO: Antisense oligonucleotide
AUC: Area under curve
CaMKII: Calcium calmodulin mediated kinase II
CD44: Cluster of differentiation 44
CDKs: Cyclin-dependent kinases
ceRNAs: Competing endogenous RNA
ctDNA: Circulating tumor DNA
CTNNBIP1: Catenin beta interactin protein 1
DLK1: Delta-like homologue 1
DLX6-AS1: Distal-less homeobox 6 antisense 1
CK1α: Casein kinase 1α
CRNDE: Colorectal neoplasia differentially expressed
CSCs: Cancer stem cells
CXCR4: C-X-C motif chemokine receptor 4
DKK-3: Dickkopf-3
DSCAM-AS1: Down syndrome cell adhesion molecule antisense RNA 1
DVL: Disheveled
EZH2: Enhancer of zeste homolog 2
FISH: Fluorescence in situ hybridization
FLVCR1-AS1: FLVCR1 antisense RNA 1
FZDs: Frizzleds
GAS5: Growth arrest-specific 5
GEO: Gene expression omnibus
GHET1: Gastric carcinoma proliferation enhancing transcript 1
GSK-3β: Glycogen synthase kinase 3β
HIF-1α: Hypoxia-inducible factor -1alpha
HNF1A-AS1: HNF1A antisense RNA 1
HNRNPL: Heterogeneous nuclear ribonucleoprotein L
HOTTIP: HOXA transcript at the distal tip
ITGB2-AS1: ITGB2 antisense RNA1
LDH: Lactate dehydrogenase
LEF: Lymphoid enhancer factor
LEF1-AS1: Lymphoid enhancer-binding factor 1 antisense RNA 1
LncRNAs: Long non-coding RNAs
LRPs: Lipoprotein receptor-related proteins
LSINCT5: Long stress-induced noncoding transcript 5
MALAT1: Metastasis-associated lung adenocarcinoma transcript 1
MDR1: Multidrug resistance 1
MEG3: Maternally expressed gene 3
MMPs: Matrix metalloproteinases
ncRNAs: Non-coding RNAs
NFAT: Nuclear factor of activated T cells
PCP: Planar cell polarity
P-gp: Glycoprotein
PKC: Protein kinase C
PLC: Phospholipase C
PP2A: Protein phosphatase 2A
qRT-PCR: Quantitative real time polymerase chain reaction
ROC: Receiver operating characteristic
ROR2: Receptor tyrosine kinase-like orphan receptor 2
RYK: Receptor-like tyrosine kinase
sFRP2: Secreted Fzd-related protein 2
sgRNA: Single-guide RNA
siRNA: Small interfering RNA
Sirt1: Sirtuin 1
SNHG1: Small nucleolar RNA host gene 1
SNHG10: Small nucleolar RNA host gene 10
SUZ12: Suppressor of zeste 12
TCF: T cell factor
TCGA: The Cancer Genome Atlas
TICs: Tumor initiating cells
TMPO-AS1: TMPO antisense RNA 1
TUFT1: Tuftelin1
TUG1: Taurine upregulated gene 1
UCA1: Urothelial carcinoma associated 1
USP47: Ubiquitin-specific peptidase 47
VEGFs: Vascular endothelial growth factor
Wif-1: Wnt inhibitory factor 1
ZEB1: Zinc finger E-box-binding homeobox 1
